# Novel insights into short-term troponin remeasurement and long-term cardiac function and structure following fulminant myocarditis

**DOI:** 10.1016/j.ijcha.2025.101759

**Published:** 2025-07-28

**Authors:** Mengmeng Ji, Luying Jiang, Zixuan Zhang, Shupeng Jiang, Houjuan Zuo

**Affiliations:** Division of Cardiology, Department of Internal Medicine, Tongji Hospital, Tongji Medical College, Huazhong University of Science and Technology, Wuhan 430030, China

**Keywords:** Fulminant myocarditis, High-sensitivity cardiac troponin I, Echocardiographic predictor, Global longitudinal strain, Long-term left ventricular function, Prognosis

## Abstract

**Background:**

Elevated serum high-sensitivity cardiac troponin (hs-cTn) levels are commonly observed in patients with fulminant myocarditis (FM) after the acute phase. This study aims to evaluate the relationship between elevated hs-cTnI levels at 30-day post-discharge and long-term cardiac structure and function.

**Methods:**

This study is a retrospective cohort study that selected FM patients hospitalized at Tongji Hospital in Wuhan from April 2016 to December 2022. All patients underwent serial monitoring of hs-cTnI levels. Patients were stratified into two groups based on hs-cTnI levels at 30 days post-discharge: the normal hs-cTnI (N-cTnI) group and the high hs-cTnI (H-cTnI) group. Left ventricular (LV) function and structure were assessed using 2-dimensional volume and speckle tracking strain echocardiography. Measurements were obtained at admission, discharge, and 6 months, 12 months, and annually thereafter post-discharge.

**Results:**

Among 95 patients with analysable echocardiographic data (median age: 33 years; 42.11 % male), the N-cTnI group demonstrated significantly higher proportions of patients meeting cardiac function criteria during follow-up compared to the H-cTnI group: LV ejection fraction (LVEF) > 50 % (95 % vs. 72 %; P = 0.003), global longitudinal strain (GLS) > 16 % (68 % vs. 36 %; P = 0.002), and LV end-diastolic dimension < 5 cm (86 % vs. 65 %; P = 0.020). Given that the primary composite endpoint occurred in only 5 patients, statistical analyses focused on secondary composite endpoints. The incidence of secondary composite endpoints was significantly higher in the H-cTnI group than in the N-cTnI group (61.91 % vs. 16.98 %; P < 0.001). Multivariable Cox regression identified elevated hs-cTnI at 30 days post-discharge (HR: 5.365; 95 % CI: 1.876–15.344; P = 0.002) and LV-GLS at discharge (HR: 0.844; 95 % CI: 0.732–0.974; P = 0.021) as independent predictors of secondary composite endpoints.

**Conclusion:**

Delayed normalization of hs-cTnI after 30 days post-discharge may predict long-term deterioration of cardiac function and structural remodeling in patients with FM.

## Graphical abstract

**Figure:**
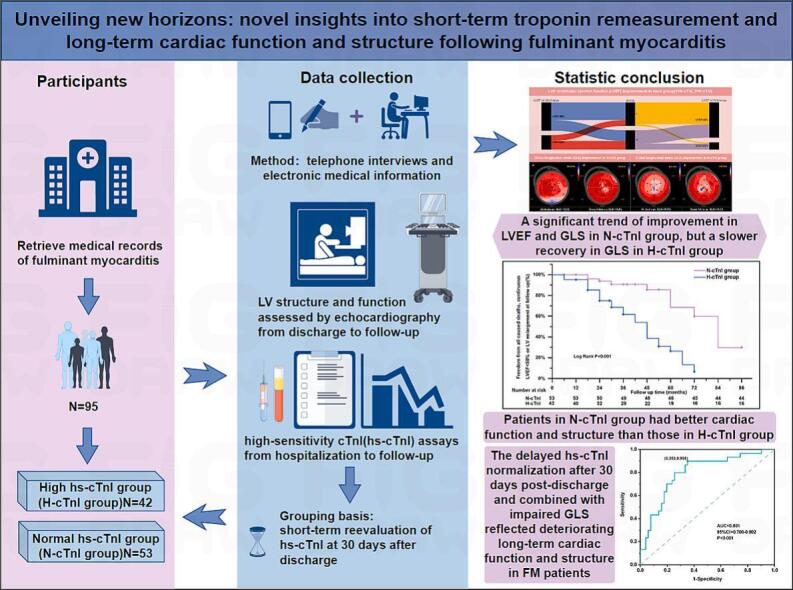

## 1 Introduction

Fulminant myocarditis (FM) is characterized by abrupt onset and severe hemodynamic compromise, frequently progressing to cardiogenic shock, multi-organ failure, and high mortality. This critical presentation constitutes one of the most life-threatening forms of myocarditis. Current clinical management necessitates early initiation of positive inotropic agents and mechanical circulatory support (MCS) [1,2]. Based on clinical experience in China, the “Life Support-Based Comprehensive Treatment Regimen” (LSBCTR), which combines MCS with immunomodulatory therapy, can significantly improve the survival rate and long-term prognosis [[3], [4], [5]]. A large cohort study of FM patients revealed that while most individuals recovered left ventricular ejection fraction (LVEF) following the acute phase, a subset of patients develop left ventricular (LV) dysfunction in the chronic phase [6]. Persistent myocardial inflammation and edema may lead to severe myocardial fibrosis and chronic myocarditis, which may ultimately impair LV function [[7], [8]]. As such, future research should focus on identifying predictive factors associated with the deterioration of cardiac function after FM.

Persistently elevated or recurrently rising levels of cardiac troponin (cTn) are indicative of ongoing or recurrent myocardial injury [[9], [10]], particularly during the early stages of FM. High-sensitivity cardiac troponin I (hs-cTnI) is especially valuable during this period, as it can more sensitively detect potential myocardial damage [[11], [12]]. It has been demonstrated that both absolute and relative changes in hs-cTnI within 24 to 48 h are strong predictors of in-hospital mortality in FM patients [13]. However, hs-cTnI levels typically decline rapidly following the initiation of timely and effective treatment. Despite this, there remains limited understanding of the long-term effects of persistently elevated hs-cTnI on cardiac structure and function. The recently updated Japanese guidelines for myocarditis recommend follow-up assessment of cTn in diagnosed cases (Class 1C), but do not specify the exact timing or frequency of these assessments [14]. Further research is necessary to determine whether hs-cTnI can predict the long-term prognosis of FM.

Given these considerations, it can be hypothesized that persistently high hs-cTnI levels during short-term follow-up may signal long-term deterioration of cardiac function and structural damage in FM patients. Therefore, this retrospective study aims to: (i) compare LV structure and function between patients with persistently elevated hs-cTnI levels after 30 days post-discharge and those with normal hs-cTnI levels; (ii) identify independent predictors of long-term cardiac function and structure in FM patients.

## 2 Method

### 2.1 Study design and participants

**Fig. 1:**
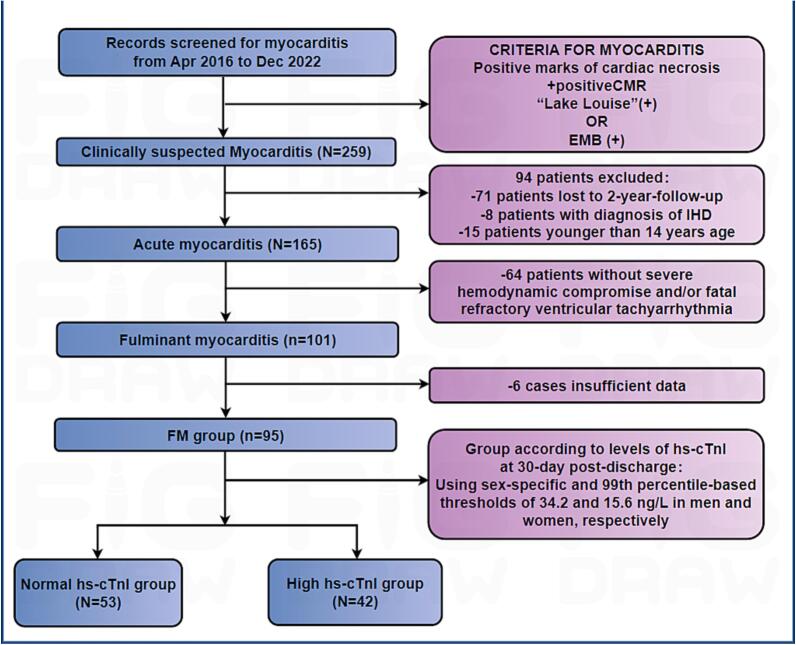
Flow diagram showing patients enrollment and exclusion.

This was a retrospective, single centre study. Tongji hospital is one of the most important mypcarditis centre in China. A total of 259 consecutive patients presenting at Tongji hospital for workup of myocarditis, identified using the International Classification of Diseases-10 (ICD-10) diagnostic code, were enrolled in the long-term follow-up between April 2016 and December 2022. Inclusion criteria of acute myocarditis was meeting one of the following conditions: 1) biopsy-proven viral myocarditis, defined by the presence of myocardial inflammation and viral genome [[15], [16]]; 2) cardiovascular magnetic resonance (CMR) performed within 5 days of initial presentation, meeting the positive “Lake-Louise” criteria for myocarditis [17]. We excluded the following patients from the study: 1) 94 patients who refused follow-up; 2) 8 patients with a prior or current diagnosis of ischemic heart disease (IHD); (3) 15 patients aged ≤ 14 years. FM were defined as severe hemodynamic compromise and/or refractory ventricular tachyarrhythmia (VT) requiring high doses of vasopressors or MCS, such as intra-aortic balloon pumps (IABPs) and/or venoarterial extracorporeal membrane oxygenation (ECMO) [18] (Fig. 1). Ultimately, 95 patients with FM were included in the study. All patients received treatment according to the “LSBCTR” [19]. Since 2018, CMR has been widely utilized at our centre for diagnosing FM, and 84 of 95 FM patients in this period underwent CMR. Endomyocardial biopsy (EMB) was performed in 56 patients as recommended by the guidelines [20]. The final diagnosis for all patients were confirmed by at least two cardiologists before inclusion in the study.

The study was conducted in compliance with the requirement for medical research ethics in the Declaration of Helsinki (2013 revision). All patients gave informed consent. And this study was approved by the Research Ethics Committee of Tongji Hospital (TJ-IRB202412013).

### 2.2 Data collection

All hs-cTnI values measured at admission were recorded and the highest one was defined as peak hs-cTnI value. In addition, hs-cTnI values were measured respectively at discharge, and within 30 days after discharge. The level of hs-cTnI is tested by ARCHITECT i2000SR (Abbott Laboratories, Chicago, U.S.). This assay has a detection range of 1.9– 50,000 ng/ L. Our study used sex-specific 99th percentile upper reference limits (URLs), with sex-specific thresholds of 34.2 and 15.6 ng/L in men and women, respectively. Patients were stratified into two groups based on 30-day post-discharge hs-cTnI levels: the normal hs-cTnI (N-cTnI) group (≤15.6 ng/L in females, ≤34.2 ng/L in males) and the high hs-cTnI (H-cTnI) group (>15.6 ng/L in females, >34.2 ng/L in males). We collected comprehensive clinical data from the electronic medical records, including demographic information, electrocardiogram (ECG) findings, vital signs, laboratory results, echocardiographic features, medications, and life-support treatments. Long-term clinical outcomes and echocardiography were assessed at 1-, 3-, 6-, and 12-months post-discharge, followed by yearly follow-up visits. All patients were followed up until May 2024.

### 2.3 Conventional 2-dimensional volume and speckle tracking strain echocardiography

All of the patients recruited into the two groups underwent a comprehensive 2D echocardiography, which was performed using a Vivid S9 Ultrasound Machine and a M5Sc transducer (GE Vingmed Ultrasound AS, Horten, Norway) at a frequency of 1 scan per 1 to 2 days from the admission till the day when the LVEF recovered before discharge. Three scans were used for the data analysis, one at admission immediately, the second one before discharge when the patients’ hemodynamic statuses were stable and LV function almost was recovered and the third one at follow-up when the newest LV function and structure were recorded.

LV structure and function were assessed by 2-dimensional volume and speckle tracking strain measurements. LV end-diastolic (LVEDD) and end-systolic diameters (LVESD), end-diastolic septal and posterior wall thickness, and LVEF using Simpson’s biplane method were taken, according to American and European recommendations [21]. Cardiac ultrasound examination was completed by evaluating LV diastolic function, measuring peak early and late diastolic velocity of the mitral inflow (respectively, E and A wave), peak septal and lateral early myocardial diastolic velocities (e′), average E/e′ ratio. Global longitudinal strains (GLS) were obtained by 2D speckle tracking echocardiography (2D STE). An experienced investigator who was unaware of the patients’ clinical information and CMR data conducted the strain analysis off-line by using EchoPAC software (version:113, 2017; GE Vingmed; Horten, Norway). The LV diameters were manually traced in each apical plane, and motion tracking was performed automatically by the software. Segmental peak systolic longitudinal strain values were averaged to achieve GLS according to AHA 17-segment LV model. According to Assessment of Left Ventricular Function by Echocardiography [22], using an LVEF cutoff value of 50 % and a GLS cutoff value of 16 %. Normal LVEDD was defined as LVEDD < 5 cm.

### 2.4 Composite endpoints

The primary composite endpoint (I type composite endpoint) was a composite of myocarditis rehospitalization or myocarditis-associated cardiovascular events, which included: readmission for HF, major arrhythmic events (eg. ventricular tachycardia/ventricular fibrillation (VT/VF)), cardiac arrest and heart transplantation (HTx). The secondary composite endpoint (II type composite endpoint) included all-cause mortality, sustained LVEF < 50 % (for > 6 months) and/or LV enlargement (LVEDD ≥ 5 cm for > 6 months).

### 2.5 Statistical analysis

The normality of distribution was assessed using the Kolmogorov–Smirnov test. Normally distributed continuous variables were described as mean ± SD and compared using the Paired *t* test, nonnormally distributed continuous variables were expressed as median and interquartile ranges (IQR) and compared using paired sample Wilcoxon rank test. Categorical variables are presented as frequencies with percentages and compared via the Pearson chi-square test or Fisher’s exact test. Kaplan-Meier (KM) curves were calculated for visualizing the cumulative survival of patients for each group. COX regression analyses were done to identify statistically significant factors attributable to fulminant myocarditis before and after adjusting for confounders. Effect-size estimates are expressed as hazard ratio (HR) and 95 % confidence interval (CI). Spearman correlation analyses were used to illustrate the relationship of significant factors, and those with pairwise correlation coefficient < 0.5 were selected as potential independent predictors for fulminant myocarditis. Comparisons of data using all these statistical tests were performed using IBM SPSS 25.0 software (SPSS Inc, Chicago, IL). Origin 2021 software was used for plotting. All statistical tests were two-sided and significance was defined as P < 0.05.

## 3 Results

### 3.1 Clinical characteristics of FM patients

Ninety-five participants (42.105 % male) were included in this study, with a median age of 33 [22,43] years. The clinical characteristics of the patients are presented in Table 1. Participants were divided into two groups: the N-cTnI group (n = 53) and the H-cTnI group (n = 42). No significant intergroup differences were observed in demographic characteristics, vital signs, clinical manifestations, or treatment regimens. At admission, proportion of patients with diabetes mellitus history, serum levels of LDH, as well as hs-cTnI levels at discharge and peak hs-cTnI levels during hospitalization, were higher in the H-cTnI group compared to the N-cTnI group.

**Table 1:** Comparison of Baseline demographic characteristics, hospital characteristics, comorbidities and treatment between the N-cTnI and H-cTnI groups.

	**Total (n = 95)**	**H-cTnI (n = 42)**	**N-cTnI (n = 53)**	**t/χ^2^/Z**	**P Value**
**Demographics**					
Age, year	33.000 [22.000, 43.000]	22.900 [21.100, 26.500]	21.500 [18.450, 25.050]	0.131	0.895
Body mass index, kg/m^2^	22.674 ±3.976	23.587 ±3.250	21.908 ±4.392	1.762	0.083
Male, n (%)	40(42.105)	18(42.900)	22(41.500)	0.017	0.895
**Vital sign**					
Systolic blood pressure,mmHg	98.000 [89.000, 111.000]	98.5000 [90.250, 111.750]	97.500 [87.500, 111.000]	0.052	0.958
Diastolic blood pressure,mmHg	63.000 [55.000, 73.000]	63.500 [57.000, 74.000]	62.000 [53.500, 71.750]	0.859	0.391
Heart rate, mean, bpm	94.000 [76.000, 112.000]	90.500 [73.750, 108.750]	100.00 [75.250, 116.000]	0.761	0.447
SpO_2_, %	98.000 [96.000, 99.000]	98.000 [96.000, 99.000]	98.000 [96.000, 99.000]	0.747	0.455
**Clinical manifestation**					
Viral prodrome, n(%)	46(48.421)	23(54.762)	23(43.396)	1.212	0.271
Fever, n(%)	55(57.895)	25(59.524)	30(56.604)	0.082	0.775
Chest tightness, n(%)	70(73.684)	31(73.810)	39(73.585)	0.001	0.980
Respiratory tract infection, n(%)	36(37.895)	13(30.952)	23(43.396)	1.542	0.214
Gastrointestinal disorders, n(%)	59(62.105)	23(54.762)	36(67.925)	1.725	0.189
Syncope, n(%)	17(17.895)	8(19.048)	9(16.981)	0.068	0.794
Arrhythmia, n(%)	39(41.053)	20(47.619)	19(35.849)	1.341	0.247
**Medical history**					
Hypertension, n(%)	7(7.368)	5(11.905)	2(3.774)	2.270	0.132
Diabetes mellitus, n(%)	5(5.263)	5(11.905)	0(0.000)	6.660	**0.010**
Hyperlipidemia, n(%)	1(1.053)	0(0.000)	1(1.887)	0.801	0.371
Smoking, n(%)	9(9.474)	4(9.524)	5(9.434)	<0.001	0.988
**ECG at admission**					
QRS interval, ms	94.000 [84.000, 118.000]	94.000 [84.000, 118.000]	93.000 [84.000, 118.500]	0.240	0.810
AV block, n(%)	24 (25.263)	11 (26.190)	13 (24.528)	0.001	0.979
VT/VF, n(%)	25 (26.316)	15(35.714)	10 (18.868)	3.404	0.065
**Laboratory datas at admission**					
Peak hs-cTnI, ng/L	26977.900 [8149.000, 49987.300]	39456.500 [23708.500, 50000.000]	19245.000 [3313.350, 37833.225]	2.672	**0.008**
hs-cTnI at discharge, ng/L	117.400 [65.000, 309.000]	253.500 [85.100, 471.100]	79.200 [28.725, 169.275]	3.765	**<0.001**
hs-cTnI at 30-day post-discharge, ng/L	16.400 [7.200, 44.300]	44.300 [27.900, 95.300]	6.050 [2.525, 8.125]	8.344	**<0.001**
CK, U/L	814.000 [285.75, 1369.000]	899.000 [463.000, 1351.000]	550.500 [216.250, 1060.000]	0.991	0.322
CRP, mg/L	19.500 [4.900, 53.200]	19.700 [9.800, 75.000]	8.900 [2.375, 27.900]	0.915	0.360
White blood cell, 10^9/L	10.145 ±6.274	10.558 ±7.591	9.818 ±5.048	0.569	0.571
AST, U/L	132.500 [72.000, 265.500]	172.000 [127.000, 319.000]	91.000 [53.000, 243.250]	1.823	0.068
ALT, U/L	52.000 [29.750, 87.500]	50.000 [37.000, 139.000]	40.500 [29.750, 166.000]	1.605	0.108
NT-proBNP, pg/mL	4821.000 [1228.000, 10015.000]	6894.000 [2441.000, 11929.000]	5992.500 [1045.250, 8551.250]	1.611	0.107
Lactic acid, mmol/L	2.345 [1.475, 3.408]	1.890 [1.410, 2.870]	3.385 [1.705, 5.675]	1.100	0.271
Creatinine, mg/dL	86.840 ±42.451	94.480 ±49.736	80.310 ±34.728	1.623	0.108
Glucose, mmol/L	7.660 [6.265, 10.198]	8.010 [6.788, 10.335]	8.320 [6.045, 11.553]	0.224	0.823
ESR, mm/H	9.000 [5.000, 16.000]	12.000 [7.000, 16.000]	6.000 [2.000, 9.000]	1.588	0.112
eGFR, ml/(min·1.73 m^2^)	91.329 ±33.508	83.905 ±35.014	98.235 ±30.861	1.981	0.051
LDH, U/L	473.500 [334.75, 731.75]	556.000 [389.000, 737.000]	340.000 [226.250, 492.500]	2.609	**0.009**
**Treatment Regimens**					
Glucocorticoid, mg	1020.000 [766.000, 1620.000]	1120.000 [765.000, 1490.000]	980.000 [706.500, 1690.000]	0.442	0.658
Days of glucocorticoid use	11.000 [8.000, 16.000]	12.000 [8.500, 15.500]	11.000 [7.000, 16.500]	1.011	0.312
IVIG, mg	60.000 [40.000, 85.000]	55.000 [42.500, 82.500]	60.000 [40.000, 90.000]	0.379	0.705
Days of IVIG treatment	6.000 [4.000, 7.000]	6.000 [5.000, 9.000]	6.000 [4.500, 7.000]	0.601	0.548
ECMO, n(%)	30(31.579)	13(30.952)	17(32.075)	0.014	0.907
IABP, n(%)	78(82.105)	34(80.952)	44(83.019)	0.068	0.794
Days of IABP use, d	4.500 [3.000,7.000]	4.000 [3.000, 7.000]	5.000 [3.000,6.500]	0.270	0.787

SpO_2,_ oxygen saturation of blood; ECG, echocardiogram; AV block, atrioventricular block; VT/VF, Ventricular tachycardia/ventricular fibrillation; hs-cTnI, high-sensitivity cardiac troponin I; CK, Creatine Kinase; CRP, C-reactive protein; ALT, alanine aminotransferase; AST, aspartate aminotransferase; NT-proBNP, N-terminal pro-B-type natriuretic peptide; ESR, erythrocyte sedimentation rate; eGFR, estimated glomerular filtration rate; LDH, lactate dehydrogenas, IVIG, intravenous immunoglobulins; IABP, intra-aortic balloon pump; ECMO, extracorporeal membrane oxygenation

### 3.2 Comparison of echocardiographic features from admission to follow up

**Fig. 2:**
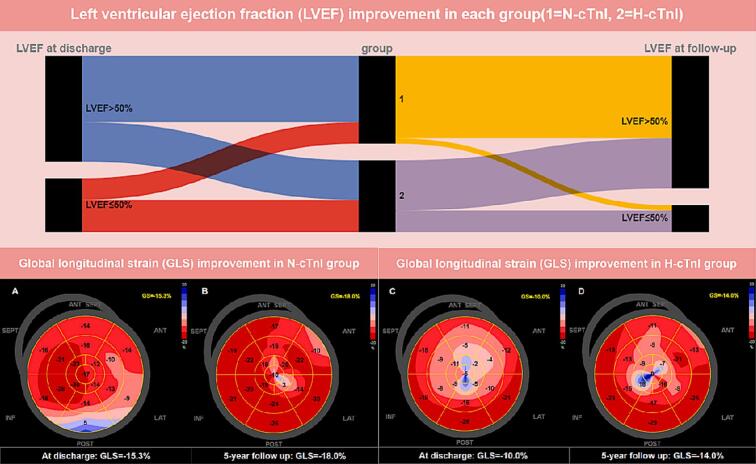
**Sankey diagram showing improvement in LVEF between the two groups from discharge to the last follow-up. Assessment of left ventricular global longitudinal strain (LV-GLS) with speckle tracking echocardiography (STE).** Example of 1 patient in two group,respectively(A-D). From the apical 4-, 2- and long-axis (APLAX) views, the GLS value is calculated as the average of the peak systolic strain of the 17 segments. The bull’s-eye plot shows more impaired GLS in the H-cTnI group.

Echocardiographic parameters at admission, discharge, and follow-up for each group are presented in Table 2. We visually demonstrated, using a Sankey diagram, that both the H-cTnI and N-cTnI groups showed a significant trend of improvement in LVEF from discharge to long-term follow-up after receiving treatment according to the “LSBCTR”. Representative images of GLS at discharge and follow-up for each group are shown in Fig. 2.

**Table 2:** Comparison of echocardiographic parameters between N-cTnI and H-cTnI at discharge and follow up.

	**N-cTnI(n = 53)**	**H-cTnI(n = 42)**	**t/χ^2^/Z**	**P Value**
**At admission**				
IVS D (cm)	0.987 ± 0.235	1.015 ± 0.244	0.561	0.576
LA D (cm)	3.000 [2.525, 3.400]	3.400 [3.000, 4.250]	3.217	**0.001**
LVEDD (cm)	4.613 ± 0.746	4.948 ± 0.586	2.380	**0.019**
E/A	1.438 ± 0.593	1.761 ± 0.782	2.093	**0.040**
E’ m/s	0.071 ± 0.030	0.060 ± 0.026	1.706	0.092
E/E’	9.735 [8.000, 12.000]	14.000 [10.000, 19.015]	3.326	**0.001**
LV ejection fraction (%)	43.000 [28.000, 49.000]	41.000 [28.500, 49.000]	0.652	0.514
GLS(%)	11.050 [6.600, 16.225]	9.100 [6.350, 14.850]	0.719	0.472
**At discharge**				
IVS D (cm)	0.946 ± 0.148	1.007 ± 0.217	1.618	0.109
IVS S (cm)	1.196 ± 0.223	1.247 ± 0.349	0.595	0.555
LVEDD (cm)	4.660 ± 0.604	4.983 ± 0.562	2.643	**0.010**
LVEDS (cm)	3.300 [3.000, 3.550]	4.000 [3.400, 5.000]	3.490	**<0.001**
End-diastolic volume (ml)	101.000 [89.000, 119.500]	137.000 [122.500, 204.000]	3.598	**<0.001**
End-systolic volume (ml)	43.000 [34.000,52.500]	70.000 [48.500, 118.000]	3.399	**0.001**
LA D (cm)	3.006 ± 0.516	3.451 ± 0.647	3.674	**<0.001**
E/A	1.360 [1.150, 2.025]	1.360 [0.865, 1.665]	0.266	0.790
E’ m/s	0.090 [0.070, 0.130]	0.060 [0.040, 0.080]	2.568	**0.010**
E/E’	8.890 [5.925, 11.610]	12.430 [9.640, 17.570]	3.142	**0.002**
LV ejection fraction (%)	58.000 [49.500, 65.000]	51.000 [40.000, 59.000]	2.743	**0.006**
GLS(%)	15.641 ± 3.837	13.647 ± 4.393	2.194	**0.031**
**Follow-up**				
IVS D (cm)	0.900 [0.800, 1.000]	0.900 [0.800, 1.000]	0.228	0.819
IVS S (cm)	1.188 ± 0.255	1.208 ± 0.396	0.286	0.776
LVEDD (cm)	4.550 [4.400, 4.800]	4.900 [4.600, 5.100]	3.150	**0.002**
LVEDS (cm)	3.100 [2.900, 3.400]	3.600 [3.300, 4.200]	4.074	**<0.001**
End-diastolic volume (ml)	108.000 [88.000, 135.000]	138.000 [113.500, 173.000]	3.859	**<0.001**
End-systolic volume (ml)	38.000 [32.000, 49.000]	56.000 [43.500, 79.000]	4.167	**<0.001**
LA D (cm)	3.116 ± 0.495	3.490 ± 0.507	3.522	**0.001**
E/A	1.375 [0.953, 1.660]	1.140 [0.855, 1.465]	1.766	0.077
E’ m/s	0.094 ± 0.029	0.077 ± 0.027	2.928	**0.004**
E/E’	8.125 [6.623, 10.473]	10.200 [7.820, 12.000]	2.700	**0.007**
LV ejection fraction (%)	61.500 [58.250, 65.000]	58.000 [48.500, 63.000]	2.550	**0.011**
GLS(%)	16.900 [14.500, 19.000]	15.500 [12.150, 16.750]	2.357	**0.018**

D,diastolic; S,systolic; IVS, interventricular septum; LVEDD, LV end-diastolic dimensions; LA, left atrium; LV,left ventricular; E, peak early diastolic mitral flow velocity; A, peak late diastolic mitral flow velocity; E’, Spectral pulsed-wave Doppler–derived early diastolic velocity from the septal mitral annulus. GLS, global longitudinal strain.

**Fig. 3:**
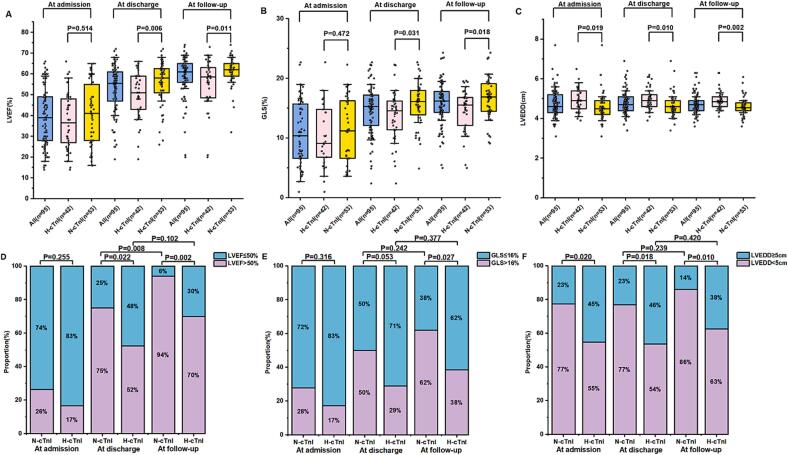
Comparison of LVEF, GLS and LVEDD at admission, discharge and follow-up in N-cTnI group with those at in H-cTnI group.

There were no statistically significant differences in LVEF and GLS between the two groups at admission. At discharge and follow-up, the N-cTnI group had significantly higher LVEF than the H-cTnI group (58.0[49.5, 65.0]% vs. 51.0[40.0, 59.0]%, P = 0.006, at discharge; 61.5[58.3, 65.0]% vs. 58.0[48.5, 63.0]%, P = 0.011, at follow-up) (Fig. 3A). Moreover, Fig. 3D illustrates that a higher proportion of patients in the N-cTnI group had normal LVEF (>50 %) compared to the H-cTnI group at discharge (75 % vs. 52 %, P = 0.022) and at follow-up (94 % vs. 70 %, P = 0.002). The N-cTnI group showed a significant improvement in the proportion of patients with normal LVEF from discharge to follow-up (75 % vs. 94 %, P = 0.008), while no significant differences were observed in the H-cTnI group (52 % vs. 70 %, P = 0.102).

In parallel with the recovery of LVEF, GLS was significantly higher in the N-cTnI group at discharge compared to the H-cTnI group (15.6 ± 3.84 vs. 13.6 ± 4.39, P = 0.031), and this difference persisted during follow-up (16.9[14.5, 19.0] vs. 15.5[12.2, 16.8], P = 0.018) (Fig. 3B). The proportion of patients with normal GLS (>16 %) was significantly higher in the N-cTnI group compared to the H-cTnI group at follow-up (62 % vs. 38 %, P = 0.027) (Fig. 3E).

LVEDD has a significant difference between the H-cTnI and N-cTnI groups at admission, discharge and follow-up (Fig. 3C). There were no significant changes in the proportion of patients with normal LVEDD (<5 cm) from discharge to follow-up in either group (H-cTnI: 54 % vs. 63 %, P = 0.420; N-cTnI: 77 % vs. 86 %, P = 0.239) (Fig. 3F).

### 3.3 Univariable and multivariable COX analysis for the prediction of II type composite endpoint

A summary of the univariable and multivariable COX regression analyses for predicting the II type composite endpoint is presented in Table 3. The I type composite outcome was observed in only 5 patients, representing 5.26 % of the sample, and therefore, was not included in the multivariable model.

**Table 3:** Univariable and Multivariable Cox Regression Analysis of composite outcome of long-term cardiac function and structure (II type composite endpoint).

**Covariates**	**Univariable**		**Multivariable**	
	**HR; 95 % CI**	**p-value**	**HR; 95 % CI**	**p-value**
Age (years)	1.025 [1.000, 1.050]	0.052		
Gender-Male (1)	1.239 [0.632, 2.430]	0.532		
Body mass index, kg/m^2^	1.055 [0.946, 1.175]	0.337		
Diabetes mellitus (1)	2.824 [0.986, 8.094]	0.053		
Hypertension (1)	3.858 [1.467, 10.150]	**0.006**	1.220 [0.320, 4.660]	0.771
White blood cell, 10^9/L	1.049 [1.007, 1.092]	**0.022**	1.031 [0.976, 1.089]	0.279
Hemoglobin, g/L	0.996 [0.977, 1.014]	0.645		
CRP, mg/L	1.001 [0.996, 1.007]	0.650		
Platelet, 10^3^/µL	1.003 [0.999, 1.007]	0.211		
Peak hs-cTnI hospitalization	1.000 [1.000, 1.000]	0.916		
Hs-cTnI at discharge	1.000 [1.000, 1.001]	0.073		
Elevated hs-cTnI at 30 days discharge(1)	4.712 [2.116, 10.490]	**<0.001**	5.365 [1.876, 15.344]	**0.002**
NT-proBNP, pg/mL	1.000 [1.000, 1.000]	0.644		
Creatinine, mg/dL	1.005 [0.998, 1.011]	0.162		
eGFR, ml/(min·1.73 m^2^)	0.991 [0.981, 1.000]	0.050		
LDH, U/L	1.000 [0.999, 1.001]	0.528		
VT/VF (1)	0.936 [0.437, 2.005]	0.865		
LA D (cm) at discharge	1.971 [1.157, 3.359]	**0.013**	0.910 [0.428, 1.934]	0.806
LVEDD (cm) at discharge	2.038 [1.296, 3.203]	**0.002**	2.069 [0.662, 6.463]	0.211
LVEDS (cm) at discharge	1.559 [0.911, 2.670]	0.105		
E/A at discharge	0.745 [0.384, 1.445]	0.384		
E/E’at discharge	1.046 [0.998, 1.096]	0.062		
LV ejection fraction (%) at discharge	0.962 [0.934, 0.990]	**0.009**	1.054 [0.996, 1.115]	0.069
GLS(%) at discharge	0.857 [0.786, 0.935]	**<0.001**	0.844 [0.732, 0.974]	**0.021**
hs-cTnI, high-sensitivity cardiac troponin I; NT-proBNP, N-terminal pro-B-type natriuretic peptide; eGFR, estimated glomerular filtration rat; LDH, lactate dehydrogenas; VT/VF, ventricular tachycardia/ventricular fibrillation; AV block, atrioventricular block; D,diastolic; S,systolic; LVEDD, LV end-diastolic dimensions; LA, left atrium; LV,left ventricular; E,peak early diastolic mitral flow velocity; A,peak late diastolic mitral flow velocity; E’,Spectral pulsed-wave Doppler–derived early diastolic velocity from the septal mitral annulus. GLS, global peak systolic longitudinal strain.				

Univariable analysis of the collected data revealed that elevated hs-cTnI levels at 30 days post-discharge, hypertension history, white blood cell, left atrium (LA) diameter (cm) at discharge, LVEDD (cm) at discharge, LVEF, and GLS at discharge were associated with II type composite endpoint (Table 3). The multivariable COX model, which included significant univariate parameters from clinical and echocardiographic domains, indicated that elevated hs-cTnI levels at 30 days post-discharge and GLS at discharge were independent predictors of II type composite endpoints.

### 3.4 Kaplan-Meier survival curve analysis and receiver operating characteristic curve analysis for II type composite endpoint of patients with FM

**Fig. 4:**
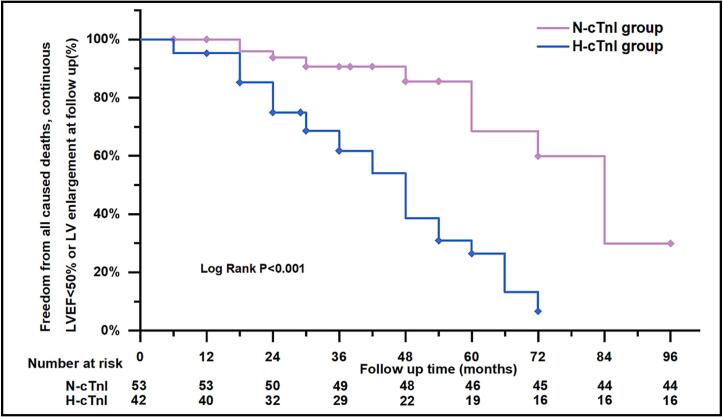
**Kaplan-Meier curve for II type composite endpoint in N-cTnI group versus in H-cTnI group.** The median survival was 48 ± 4.704 months, 95 %CI[38.781,57.219] in H-cTnI group. The median survival was 84 ± 8.889 months, 95 %CI [66.578,101.422] in N-cTnI group. There were significant differences in II type composite endpoint between H-cTnI and N-cTnI groups (logrank P < 0.001).

**Fig. 5:**
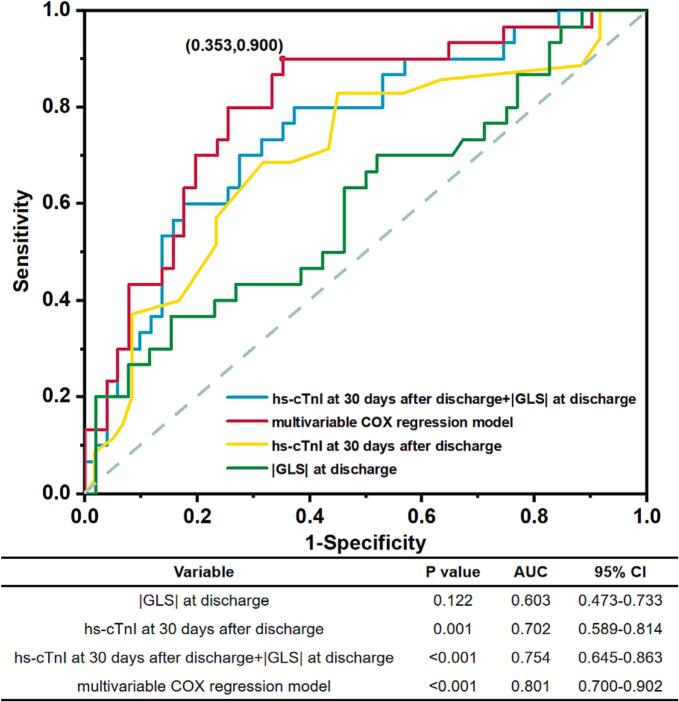
**Receiver operating characteristic curve (ROC) analysis for the value of hs-cTnI to predict the II type composite endpoint at follow up.** hs-cTnI at 30-day post-discharge could predict secondary composite endpoints incidence (P = 0.001). Multivariable COX regression model could predict occurrence of II type composite endpoint with a higher sensitivity 90.00 % and higher specificity of 64.70 %.

The long-term primary and secondary composite endpoints during the follow-up period are reported in Supplemental Table 1. The mean follow-up duration for all patients was 36.99 ± 19.89 months. In the K-M survival curve analysis for the I type composite endpoint, normal levels of hs-cTnI were significantly associated with a better prognosis (log-rank P = 0.012, Supplemental Fig. 2). Similarly, the K-M survival curve showed a significantly higher occurrence of II type composite endpoint in H-cTnI group (log-rank P < 0.001, Fig. 4). An ROC curve was generated to determine the cutoff values and assess the elevation of hs-cTnI in predicting deteriorating cardiac function in FM (Fig. 5). The results indicated that the predictive value of hs-cTnI at 30 days post-discharge and GLS at discharge (AUC = 0.754; 95 % CI, 0.645–0.863) for II type composite endpoint in FM. The largest AUC (0.801, sensitivity = 0.900, specificity = 0.647, P < 0.001) was observed when relevant factors from the multivariable COX regression model were included.

## 4 Discussion

The main finding of this retrospective cohort study is that beneficial structural and functional alterations of LV following discharge from the hospital in FM patients are often characterized by rapid normalization of hs-cTnI levels, manifested as reduced LV volumes and increased in LVEF. Furthermore, abnormally elevated hs-cTnI levels during short-term follow-up (30 days post-discharge) serve as an important independent predictor of worsening cardiac function and structure in FM patients. This provides additional risk stratification information for FM patients. Additionally, abnormally elevated hs-cTnI levels were also predictive of all-cause mortality. Multivariable analysis further showed improved LV-GLS is an independent predictor of the secondary composite endpoint, negatively correlated with LV dilatation and worsening LV function.

The high myocardial specificity and clinical sensitivity of hs-cTnI for myocardial injury are well established [23]. Previous studies have shown no significant correlation between elevated cTnI levels and incomplete recovery of cardiac function and structure in FM patients, both in the short and long term [[24], [25]], but some studies have reported opposite results [10,13,26]. The study by Waleed Albuali et al. showed significantly high predictive validity of troponin levels in relation to the severity of myocarditis in children (P = 0.001) [27,28]. Notably, a study of acute myocarditis reported that short-term reevaluation showed significant incremental prognostic value compared to baseline evaluation (baseline model vs. 6-month model: AUC 0.79 vs. 0.90, P = 0.03) [29], providing novel insights into prognosis evaluation for FM. Our study aims to provide additional evidence regarding the relationship between follow-up assessment of cTnI elevations and LV dysfunction in FM.

From a pathophysiological perspective, direct cardiac damage during the acute phase of FM leads to the release of hs-cTnI [30]. Subsequent increased cell death in the myocardium triggers inflammatory responses, further elevating hs-cTnI levels and worsening the condition [31]. Chen et al. suggested that changes in TnI levels reflect the potential recovery of stunned myocardium in FM, with lower TnI levels indicating a relatively mild degree of necrosis and inflammation, leading to early functional recovery [32]. A significant association has been reported between hs-cTnI concentration and rates of all-cause death or hospitalization for HF [33]. Elevated hs-cTnI levels could reflect ongoing subclinical myocardial damage or micro-infarctions, independent of acute ischemic injury [34]. While the evidence for this is debated.

Most previous studies have shown that reduced LVEF (<50 %) and longer LVEF recovery times during hospitalization are risk factors for cardiac function impairment after FM discharge [[35], [36]]. In contrast, Ammirati et al. demonstrated that reduced LVEF at admission without severe hemodynamic compromise did not correlate with prognosis, suggesting that clinical presentation with severe hemodynamic compromise is the primary determinant of both short- and long-term prognosis [37]. This debate arises because cardiac impairment at admission may involve both actual tissue necrosis and reversible damage due to inflammation. To address this, we evaluated LVEF and GLS at discharge to avoid potential interference from reversible hemodynamic instability and acute inflammatory response, thus enhancing the reliability and stability of our findings. Our study confirmed that LVEF and GLS at discharge were associated with long-term secondary composite endpoints. Specifically, GLS served as an independent predictor of this endpoint and its improvement was inversely associated with LV dilatation and worsening LV function. During follow-up, both groups showed a trend toward higher LVEF and GLS, but the H-cTnI group demonstrated slower recovery in GLS, resulting in significantly lower GLS compared to the N-cTnI group. These findings are consistent with recent experimental studies, which found that impairment in GLS correlates with systolic and diastolic LV function and cardiac biomarkers but remains unchanged over time, even after clinical recovery [[38], [39]]. This may indicate that a decreased GLS represents subtle myocardial changes that persist after clinical recovery. Notably, previous studies have shown that myocardial strain has a higher sensitivity than conventional echocardiography, and therefore, may be an important tool to detect early subclinical cardiac dysfunction [[40], [41], [42]].

Based on our results, patients with in H-cTnI group had higher E/E’ ratios and lower E’ at discharge than the N-cTnI group at following suggesting higher LV filling pressures. LV diastolic filling parameters (especially LVEDD) improvements persisted over time in patients in two groups, but remains statistic differences. It is well known that FM is associated with severe and refractory HF [1]. Increased preload (diastolic wall stress) is a key feature of the failing heart. Both clinical and experimental studies suggest it may initiate troponin release [[43], [44]]. LV hypertrophy and raised LV preload could lead to increased cTn release as a result of myocardial strain and myocardial O^2^ supply–demand mismatch [45]. Thus myocardial strain consequent to increased LV load could present another mechanism by which cTn is elevated. In turn, cTnI level could be the valuable predictor for LV strain injury.

Given that fulminant myocarditis is a complex and often fatal disease, the role of any single factor in its pathogenesis is likely small when assessed in isolation. Our multivariable COX analysis revealed that adding clinical features and echocardiographic predictors (LA D at discharge, LVEDD at discharge, LVEF at discharge) significantly improved prediction capability (AUC: 0.754 vs. 0.801). This result underscores the importance of assessing short-term hs-cTnI levels and echocardiographic improvements early on for the prognostic management of FM patients.

Tracking hs-cTnI changes during follow-up in FM patients is important for understanding the disease and providing better treatment. If hs-cTnI levels do not decrease promptly, we recommend close follow-up of hs-cTnI levels. Although the risk is continuous, establishing prognostic thresholds is relevant in clinical practice to guide physicians in patient assessment, risk stratification, and consideration of therapies to mitigate further events in high-risk patients. Future studies should expand the study population to validate our findings and investigate whether targeted interventions can reduce cardiovascular risk in FM patients, particularly in those with persistent elevations in hs-cTnI. Additionally, patients exhibiting left atrial enlargement, LV enlargement, and/or dysfunction at discharge represent high-risk groups that warrant frequent reevaluations. These factors are critical for enhancing prognostic stratification and guiding individualized, long-term management strategies for FM patients.

## 5 Conclusions

Hs-cTnI serves as a key indicator of the extent of myocardial damage in FM. The delayed hs-cTnI normalization within 30 days post-discharge may reflect ongoing myocardial injury or inflammation and is associated with deteriorating long-term cardiac function and structure in FM patients, highlighting the importance of close monitoring of hs-cTnI levels in the post-discharge period.

## 6 Limitations

Our study should be interpreted in the context of its limitations. First, as a single-center study, our study population and results may not be directly generalizable to all other healthcare settings or broader populations. More studies should be considered to implement to validate the findings in a larger external cohort. Secondly, we did not perform EMB in all patients. Therefore, we used the term FM and data obtained from this study population may not be able to apply to lymphocytic myocarditis completely. However, it must be noted that EMB can’t be completed in many centres in the real world. The percentage of EMB in many reports of FM was also low [10,26,46]. Finally, some outcomes after the follow-up period could be missed if the follow-up terminates prematurely, which might indicate selection bias (attrition bias).

## 7 Sources of Funding

The project was supported by a grant from the Natural Science Foundation of China (No. 81873535) and the Natural Science Foundation of Hubei Province (No. 2020CFB573).

## 9 Availability of data and materials

The information and data of the study population were acquired from Hospital Information System and were recorded manually in EXCEL to form the database. The datasets analyzed during the current study are not publicly available due to the protection of the individual privacy but are available from the corresponding author on reasonable request.

## Author contributions

MM.J. and LY.J. was responsible for the initial draft of the manuscript and contributed to the study design. MM.J., LY.J., ZX.Z., SP.J., and HJ.Z. contributed to data acquisition, data analysis, and interpretation of data for the work. MM.J., LY.J. and HJ.Z. contributed to revising the manuscript critically for intellectual content and approved the final version for publication. All authors reviewed the manuscript critically for intellectual content and have read and approved the final manuscript.

## CRediT authorship contribution statement

**Mengmeng Ji:** Writing – review & editing, Methodology. **Luying Jiang:** Writing – review & editing, Methodology, Investigation, Conceptualization. **Zixuan Zhang:** Visualization, Software, Investigation, Formal analysis. **Shupeng Jiang:** Software, Resources, Data curation. **Houjuan Zuo:** Writing – review & editing, Project administration, Funding acquisition, Conceptualization.

## 8 Ethics approval and consent to participate

The study protocol was approved by the ethics review board of Tongji Hospital, Huazhong University of Science and Technology (approval number:TJ-IRB202412013). The study was conducted in accordance with the Declaration of Helsinki and the principles of good clinical practice. Informed consent was obtained from all participants discharged.

## Declaration of competing interest

The authors declare that they have no known competing financial interests or personal relationships that could have appeared to influence the work reported in this paper.

## Appendix A Supplementary data

Supplementary Fig. 1 Kaplan-Meier curve for I type composite endpoint in N-cTnI group versus in H-cTnI group.
