# Supplementary figures and images for: Novel insights into short-term troponin remeasurement and long-term cardiac function and structure following fulminant myocarditis
Source: Int J Cardiol Heart Vasc. 2025 Jul 28;60:101759. doi: 10.1016/j.ijcha.2025.101759 (PMC12329257; doi:10.1016/j.ijcha.2025.101759)

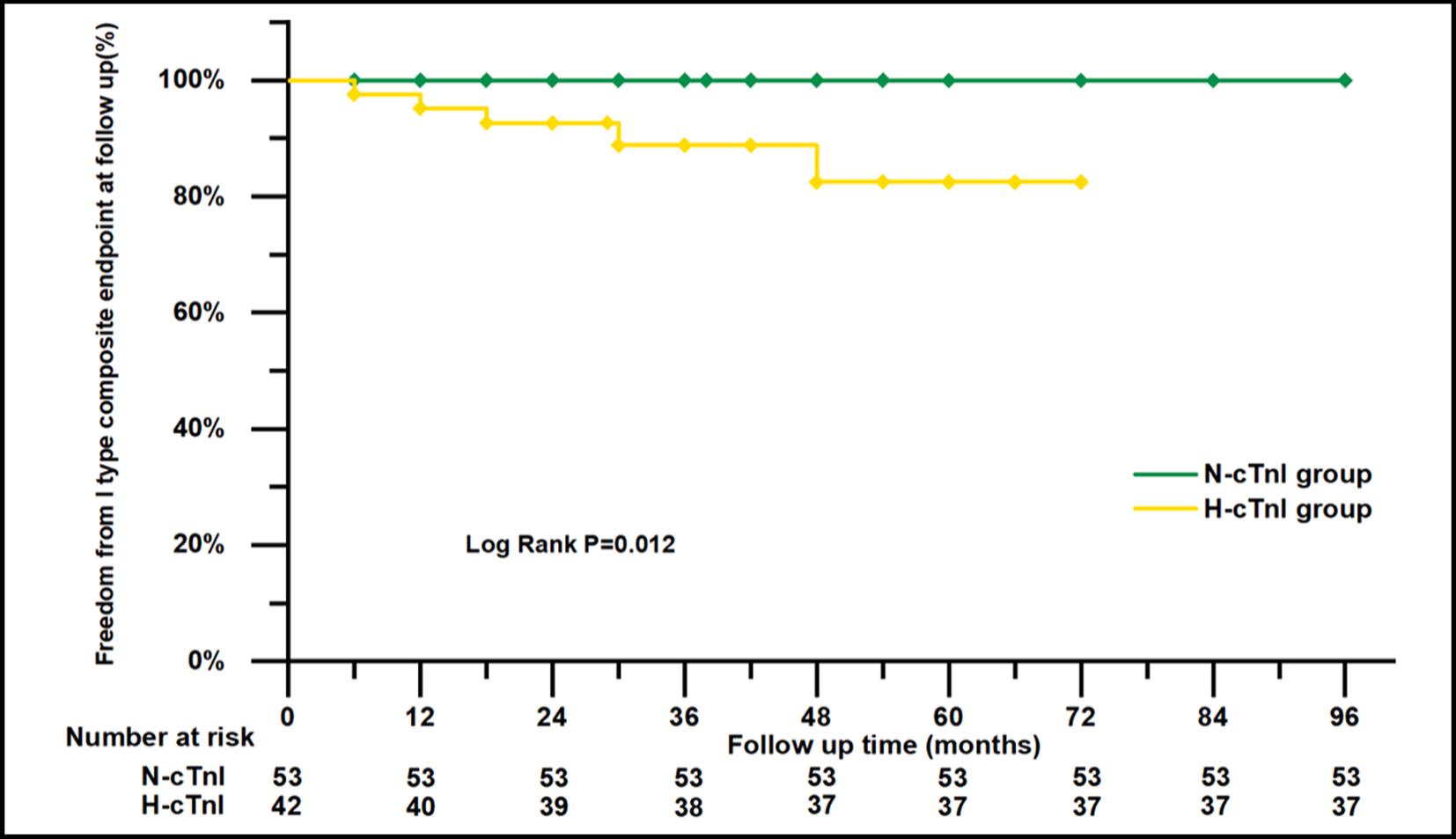

Supplement: Supplementary Fig. 1 — Kaplan-Meier curve for I type composite endpoint in N-cTnI group versus in H-cTnI group. [file mmc2.jpg]
