# Supplementary material for: Novel insights into short-term troponin remeasurement and long-term cardiac function and structure following fulminant myocarditis
Source: Int J Cardiol Heart Vasc. 2025 Jul 28;60:101759. doi: 10.1016/j.ijcha.2025.101759 (PMC12329257; doi:10.1016/j.ijcha.2025.101759)
Supplement: Supplementary Data 1 [file mmc1.docx]

| **Supplemental Table 1. Major outcome events during long-term follow-up** | | |  | | |
| --- | --- | --- | --- | --- | --- |
| **Outcome** | **Total (n=95)** | **N-cTnI (n=53)** | **H-cTnI (n=42)** | **t/χ²/Z** | **P Value** |
| I type composite endpoint, n% | 5 (5.263) | 0 (0.000) | 5 (11.905) | 4.089 | **0.010** |
| Myocarditis rehospitalization, n% | 3 (3.157) | 0 (0.000) | 3 (7.143) |  |  |
| Hospitalization HF, n% | 1 (1.053) | 0 (0.000) | 1 (2.381) |  |  |
| HT(x), n% | 1 (1.053) | 0 (0.000) | 1 (2.381) |  |  |
| II type composite endpoint, n% | 35 (36.842) | 9 (16.981) | 26 (61.905) | 14.258 | **<0.001** |
|  | | | | | |
